# Case Report: Endoscopic submucosal dissection revealed isolated gastric metastasis from primary bladder urothelial carcinoma: clinicopathological analysis and literature review

**DOI:** 10.3389/fmed.2025.1591783

**Published:** 2025-08-22

**Authors:** Famei Xu, Guihua Li, Guihong Qiu, Hongbo Yu, Qing Yin, Fabao Xu, Tianzi Jian

**Affiliations:** ^1^Department of Pathology, Zibo Central Hospital, Zibo, China; ^2^Department of Cadre Health, Zibo Central Hospital, Zibo, China; ^3^Department of Pathology, Zhoucun People’s Hospital, Zibo, China; ^4^Department of Ophthalmology, Qilu Hospital, Shandong University, Jinan, China; ^5^Department of Hematology, Qilu Hospital, Shandong University, Jinan, China

**Keywords:** urothelium carcinoma, gastric metastases, oligometastasis, treatment, endoscopic submucosal dissection

## Abstract

A 73-year-old male was admitted to our department with complaints of upper abdominal distension, accompanied by dull pain and belching for more than 10 days. Gastroscopy revealed a broad-based raised lesion, approximately 1.0 cm in diameter, on the anterior wall of the gastric body, with a central star-shaped depression, erosion, and surrounding congestion. Endoscopic ultrasonography showed a lesion on the lower anterior wall of the gastric body involving the submucosal layer, with a subsequent biopsy indicating cancer. Notably, the patient had undergone cystectomy seven months earlier, with a pathological diagnosis of an invasive high-grade nested variant of urothelial carcinoma staging pT2N0Mx. An endoscopic submucosal dissection (ESD) was eventually conducted for diagnostic purposes. A gross examination of the specimen revealed a superficial elevated tumor measuring 1.2 × 1 × 0.3 cm, with a central depression, a grey-white cut surface, and firm texture. Microscopically, the tumor cells exhibited architectural and cytomorphological features resembling those of a bladder tumor. Immunohistochemical staining was positive for GATA-3, 34βE12, CK7 and negative for p63, which were consistent with those observed in bladder tumors. Based on the clinicopathological features and medical history, a diagnosis of gastric oligometastatic urothelial carcinoma was made. Following ESD, the patient received four cycles of gemcitabine chemotherapy and showed no sign of recurrence at the 41-month follow-up.

## Introduction

Bladder cancer is one of the most common malignancies of the urinary system, accounting for approximately 3.0% of all new cancers and 2.1% of cancer-related deaths (1). It includes non-muscle invasive bladder cancer and muscle-invasive bladder cancer. The incidence is slightly higher in males than in females, and two-thirds of cases are non-muscle invasive bladder cancer, characterized by a high recurrence rate but low mortality. The five-year survival rate for patients with muscle-invasive bladder cancer is only 60% (2–4), and 10 to 15% of these patients present with metastases at the time of recurrence (5). Regional lymph nodes, lungs, liver, and bone are the most common sites of metastasis in bladder cancer. Metastasis to other internal organs is rare, and gastric metastasis is extremely uncommon. According to a literature search, only about 10 cases of bladder cancer metastasizing to the stomach have been reported to date. These include eight cases identified by Wallmeroth et al. (6) in an autopsy study of 367 patients with bladder cancer, and a case report describing diffuse gastric metastasis resulting in linitis plastica (7). Currently, systemic chemotherapy is the standard treatment for metastatic bladder cancer. Nevertheless, most patients experience disease progression despite chemotherapy (8, 9). In recent years, although the feasibility and effectiveness of surgical treatment for metastatic bladder cancer have not been fully established, reports on the surgical management of oligometastatic bladder cancer have gradually increased. The present paper presents a rare case of gastric oligometastatic bladder cancer (postoperative pathological staging after cystectomy pT2N0Mx) identified in endoscopic submucosal dissection (ESD) specimen and summarizes its clinicopathological features and treatment approach to provide insights into its diagnosis and management.

## Case presentation

**Figure 1:**
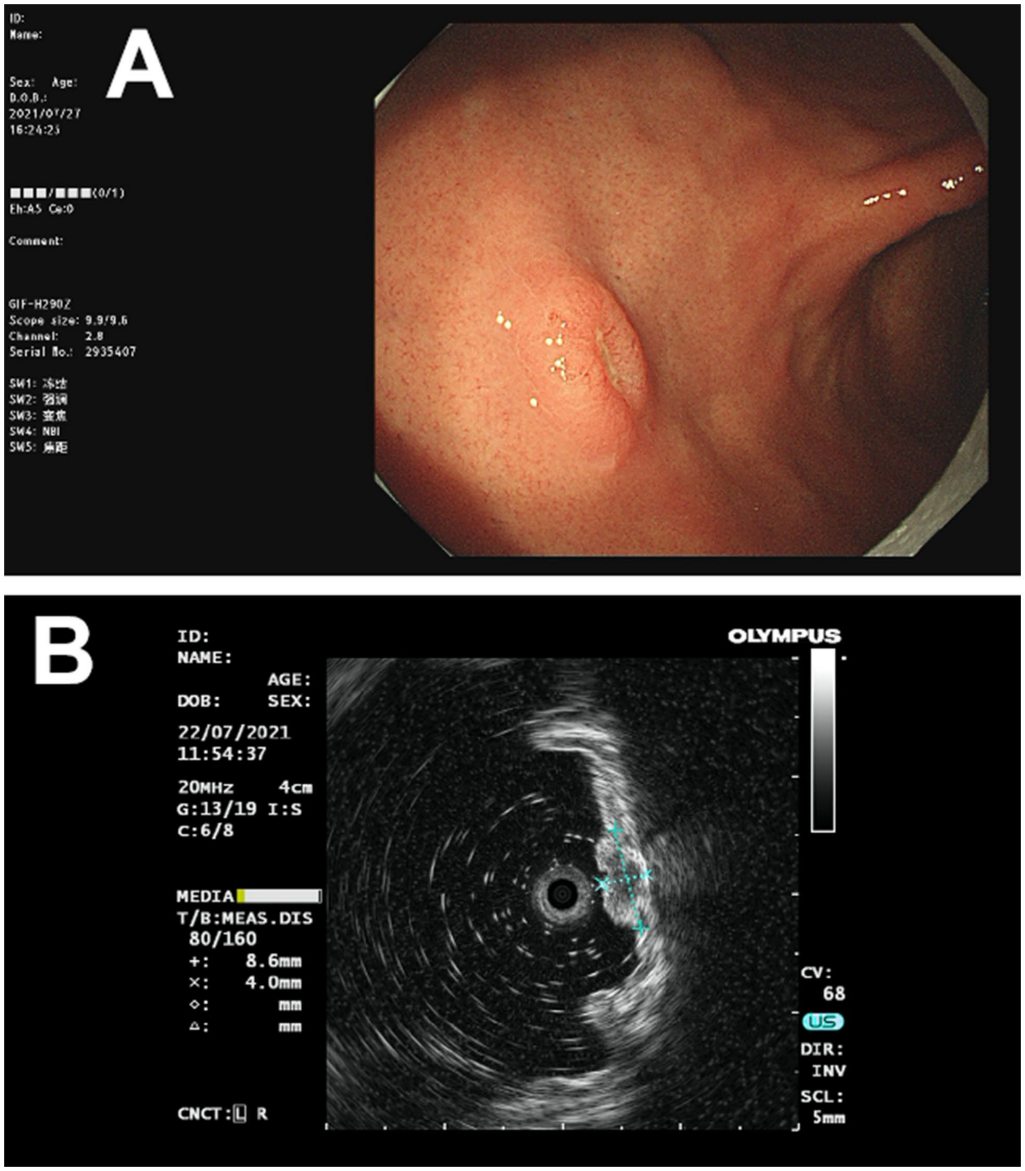
The images of gastric lesions obtained through endoscopy and endoscopic ultrasound. **(A)** Gastroscopy revealed a broad-based raised lesion, approximately 1.0 cm in diameter, on the anterior wall of the gastric body, with a central star-shaped depression, erosion, and surrounding congestion. **(B)** Endoscopic ultrasonography showed a lesion on the lower anterior wall of the gastric body, invading the submucosal layer.

**Figure 2:**
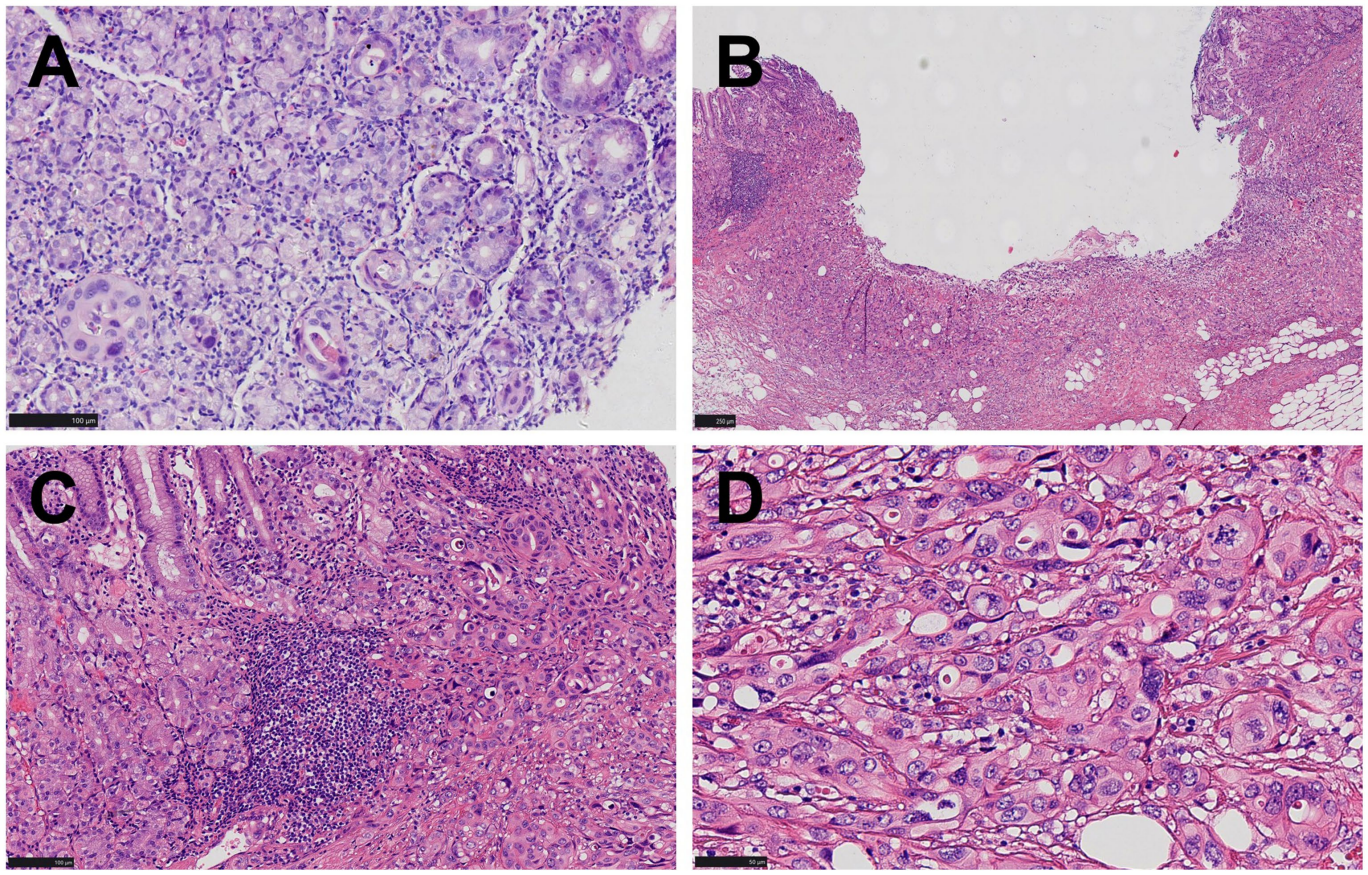
Pathological manifestations of gastric lesions. **(A)** Only rare foci of glandular structures demonstrating cellular atypia are identified in the biopsy specimen (HE, ×200). **(B)** A superficial concave lesion of gastric mucosa and submucosa (HE, ×100). **(C)** Nests of dysplastic cells proliferated in the mucosa and submucosa, lacking glandular structures. Chronic inflammatory cell infiltration and focal lymphoid hyperplasia were observed in the mucosa around the tumor, and no atrophic gastritis, gastric intestinal metaplasia, or dysplasia was observed (HE, ×200) **(D)** Cells were relatively large and polygonal, with moderate cytoplasm, large round or oval nuclei, coarse chromatin, with some exhibiting a clumped pattern and distinct nucleoli, while mitotic figures were readily observed (HE, ×400).

The patient, a 73-year-old male, was admitted to our department (July 2021) with complaints of upper abdominal distension for over 10 days, accompanied by dull upper abdominal pain and belching, which occurred both on an empty stomach and after meals, along with constipation. Gastroscopy revealed a broad-based elevated lesion approximately 1.0 cm in diameter on the anterior wall of the gastric body, with a central star-shaped depression, erosion, and surrounding congestion (Figure 1A), along with hiatal hernia, reflux esophagitis (LA-B), and chronic non-atrophic gastritis. In order to determine the depth of tumor invasion, an endoscopic ultrasound examination was performed. Endoscopic ultrasonography showed a lesion on the lower anterior wall of the gastric body invading the submucosal layer (Figure 1B). This patient underwent a gastroscopy biopsy at another hospital about 10 days ago (June 2021), which revealed mild chronic non-atrophic gastritis, nests of dysplastic cells in the gastric body mucosa, suggesting cancer (Figure 2A), with no diagnosis of adenocarcinoma, no assessment of the degree of differentiation, and no determination of whether it is a primary tumor of the stomach. Of note, the patient had previously undergone radical cystectomy for bladder cancer seven months earlier (December 2020) in the same hospital, staging pT2N0Mx. No evidence of metastatic disease was observed. There was no adjuvant treatment after bladder cancer surgery. Consequently, diagnostic ESD resection was performed (July 2021) in our hospital.

### Gross specimen examination following ESD

A piece of gray-red mucosa from the lower gastric body, measuring 2.6 × 2 cm with a thickness of 0.4 cm. In the central area, a gray-red superficial elevated lesion measuring 1.2 × 1 × 0.3 cm was identified, featuring a central depression and a firm gray-white cut surface. The peripheral cutting margin ranged from 0.1 to 0.8 cm, with the surrounding mucosa appearing gray-red and of medium texture.

### Pathological diagnosis

Microscopic examination revealed nests of dysplastic cells proliferating within the mucosa and submucosal layer. These cells were tightly packed, with no obvious glandular structures and sparse stroma. The relatively large polygonal cells exhibited moderate cytoplasm, bicolored appearance, large round or oval nuclei, coarse chromatin, some with clumped chromatin, distinct nucleoli, and frequent mitotic figures, including atypical mitosis (Figures 2B–D). The surrounding mucosa showed chronic inflammation and focal lymphoid hyperplasia without dysplasia (Figure 2C).

**Figure 3:**
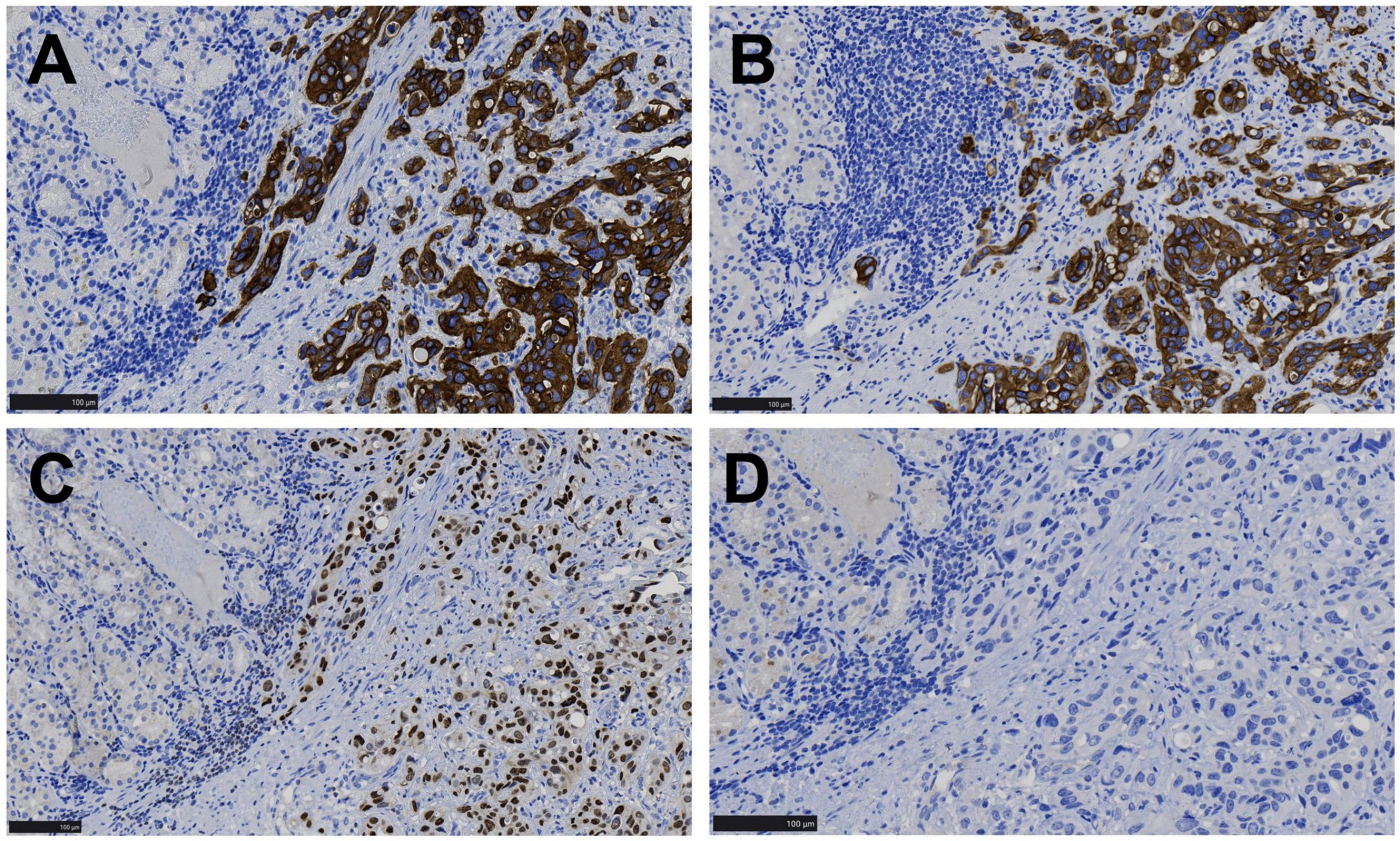
Immunohistochemical images of gastric lesions. **(A,B)** Tumor cells displayed CK7 and 34βE12 positivity in the cytoplasm, while the surrounding gastric mucosal gland epithelium was negative (EnVision, ×200). **(C)** Tumor cells showed nuclear GATA-3 positivity, while the surrounding gastric mucosal gland epithelium was negative (EnVision, ×200). **(D)** Both tumor cells and the surrounding gastric mucosal gland epithelium were negative for p63 (EnVision, ×200).

Immunohistochemical staining showed that the tumor cells were positive for CKAE1/AE3, CK8/18, CK7, 34βE12, and GATA-3 (Figures 3A–C), while negative for p63 (Figure 3D), Vimentin, MUC5AC, MUC6, Hep-1, AFP, CK20, CDX2, MUC2, CD10, CK5/6, CD56, Syn, and CgA. MLH1, PMS2, MSH2, and MSH6 were strongly positive (+++95%), C-erbB-2 was negative (0), p53 was moderately positive (++70%), and Ki-67 showed a proliferative index of 70%.

**Figure 4:**
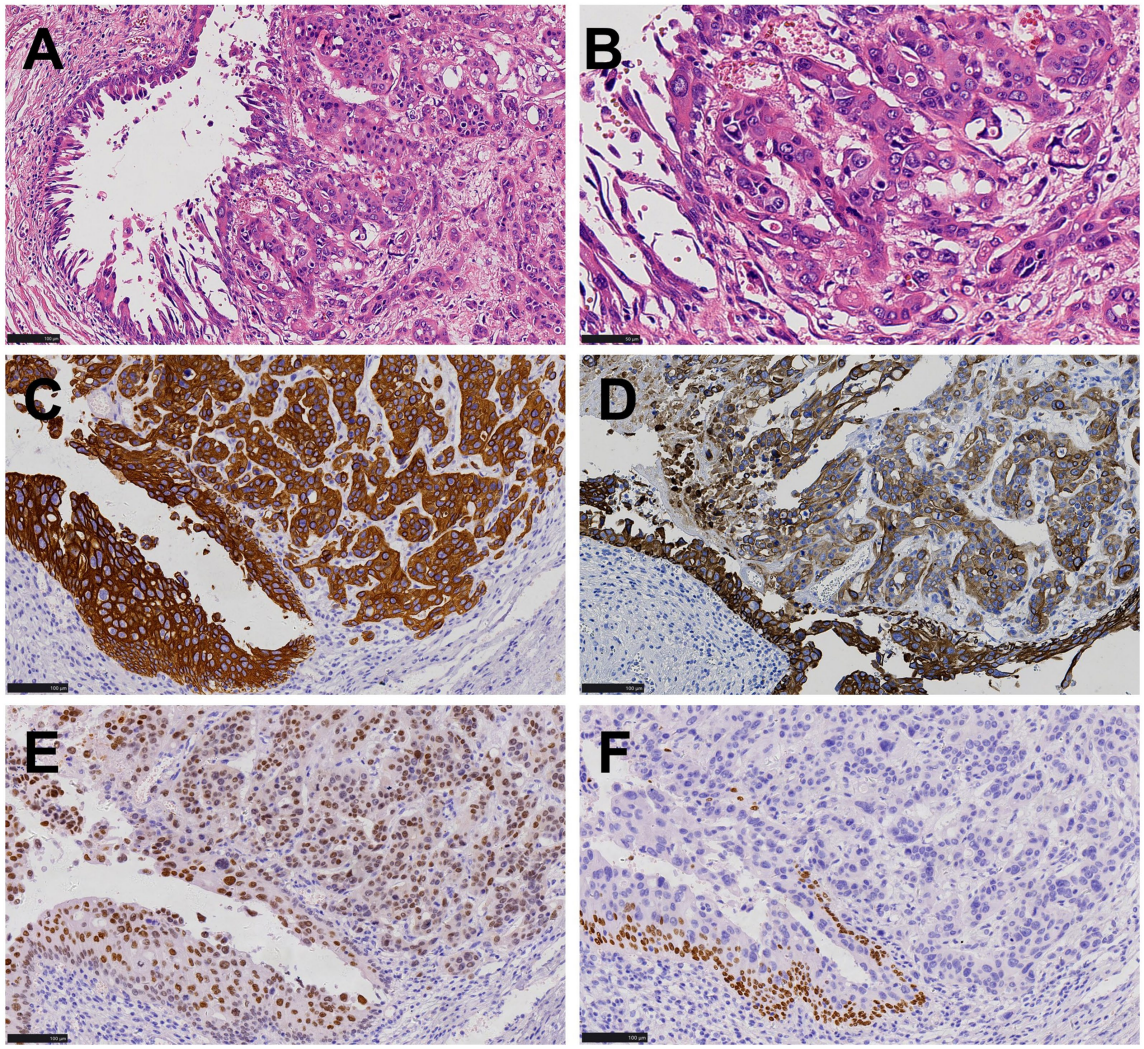
Histopathological and immunohistochemical images of bladder cancer. **(A,B)** The arrangement structure and cell morphology of bladder tumors were consistent with that of stomach tumors as shown in Figure 2, urothelial carcinoma *in situ* was present in the surrounding mucosa (HE, ×200, ×400). **(C,D)** Both tumor cells and peripheral mucosal urothelial carcinoma in situ were positive for CK7 and 34βE12 (EnVision, ×200). **(E)** Tumor cells were positive for GATA-3 (EnVision, ×200). **(F)** Tumor cells were negative for p63 (EnVision, ×200).

Subsequently, we reviewed the pathological features of the bladder tumor in this patient and found that the arrangement structure, cell morphology (Figures 4A,B) and immunohistochemical expression (Figures 4C–F) of the bladder tumor were exactly the same as those of the stomach tumor.

The ESD specimen from the lower gastric body revealed poorly differentiated carcinoma confined to the mucosa and submucosa. Based on the immunohistochemical findings and clinical history, the lesion was diagnosed as metastatic high-grade urothelial carcinoma originating from the bladder. The tumor measured 1.2 × 1 × 0.3 cm, with an infiltration depth reaching up to 800 microns into the submucosa. The resection margins were negative. The surrounding mucosa exhibited chronic inflammation with focal lymphoid hyperplasia.

### Treatment and follow-up

The patient underwent four cycles of gemcitabine chemotherapy and received treatment with medications such as omeprazole and dexime following the ESD procedure. His abdominal symptoms disappeared and has been followed up for 41 months without any signs of recurrence.

## Discussion

Metastasis of gastric cancer to other organs is relatively common, while metastasis of malignant tumors to the stomach is extremely rare. It has been reported that metastatic tumors account for only 0.2–0.7% of all gastric malignant tumors (10, 11). These data are primarily based on case reports and small case series (12), with the most common malignant tumors that metastasize to the stomach being malignant melanoma, breast cancer, lung cancer, esophageal cancer, and renal cell carcinoma (13, 14). Only about 10 cases of bladder cancer metastasis to the stomach have been reported, including postmortem cases, as detailed in Table 1 (6, 7, 15, 16). A case of plasmacytoid urothelial carcinoma was initially treated with transurethral resection of the mass and Bacille Calmette-Guerin (BCG) injection into the bladder. Six years later, antral and systemic lesions were discovered, and a gastric biopsy was mistakenly diagnosed as poorly differentiated adenocarcinoma of the stomach. The patient underwent total gastrectomy after neoadjuvant chemotherapy (16). Postoperative pathology revealed that the gastric tumor was metastatic bladder cancer. It is observed that the diagnosis of bladder cancer metastasis to the stomach can be challenging, and misdiagnosis is common. Herein, we report the case of a 73-year-old man with a single metastatic gastric lesion that appeared seven months after surgery for bladder cancer.

**Table 1:** Summary of patient characteristics in studies involving patients with bladder urothelial carcinoma metastasizing to the stomach.

Studies	Patients	Primary lesion	Gastric metastasis	Treatment	Overall survival
Hong et al. (7)	1	T4	Cardia to the antrum	Chemotherapy + radiation therapy	NA
Kim et al. (15)	1	Solitary	Body	Chemotherapy	12 months
Nabbout et al. (16)	1	NA	Body and antrum	Gastrectomy	>96 months
Wallmeroth et al. (6)	8	NA	NA	NA	NA
Xu et al. (n.d., unpublished data)	1	Solitary, T2	Body	Cystectomy, ESD + chemotherapy	41 months

NA, not applicable.

Clinical symptoms of gastric metastatic tumors are often nonspecific, typically presenting as upper abdominal pain, nausea, vomiting, loss of appetite, gastrointestinal bleeding, anemia, and other symptoms (17). In the present case, the patient presented with abdominal distension, accompanied by dull pain in the upper abdomen, belching, and constipation. In addition to the tumor, the patient’s endoscopic diagnosis also revealed hiatal hernia, reflux esophagitis (LA-B), and chronic non-atrophic gastritis. The abdominal symptoms of this patient might be the result of the combined effects of the above-mentioned lesions.

Metastasis of malignant tumors to the stomach primarily occurs through hematogenous spread, with lesions typically implanting in the gastric submucosa. These lesions often present as one or more discrete submucosal nodules under endoscopy, though they may also form large masses or ulcerations. In rare cases, the tumors infiltrate and expand through the mucosa, submucosa, and serosa, leading to the formation of linitis plastica (12, 17, 18). Here, gastroscopy revealed a single raised nodule on the anterior wall of the gastric body, with a basal diameter of approximately 1.0 cm. This morphological feature could also occur in early gastric cancer, neuroendocrine tumors, or small gastrointestinal stromal tumors, among others. Therefore, the endoscopic appearance is not sufficiently specific to make a definitive diagnosis, and a biopsy is necessary for pathological examination.

However, even with endoscopic biopsy, the diagnostic rate for gastric metastatic cancer is only 15.6% (10). Key contributing factors may include the following: Firstly, the small tissue sample contained only minimal tumor tissue, restricting comprehensive analysis. Secondly, the tumor’s histological appearance may have changed following metastasis, complicating diagnosis. Thirdly, detailed medical background from clinicians was lacking, hindering accurate interpretation. Finally, the case demanded a high level of diagnostic acumen from pathologists, particularly in differentiating subtle features. The biopsy specimen posed significant diagnostic difficulties due to its scant tumor content and a misleading, atypical glandular architecture that obscured definitive classification. Immunohistochemical profiling was unfeasible given the low tumor cell yield. The biopsy indicated mild chronic non-atrophic gastritis, alongside dysplastic cell nests in the gastric body mucosa-suggestive of malignancy. However, no definitive diagnosis of adenocarcinoma was rendered, nor was tumor differentiation or primary origin (gastric vs. metastatic) determined.

After ESD intervention, microscopic examination revealed that the tumor cells exhibited distinct morphology and structure, with polygonal nests of tumor cells displaying characteristics of both cancer and sarcoma. There was an absence of glandular arrangements. Upon reviewing the patient’s medical history, it was noted that the patient had been diagnosed with urothelial carcinoma seven months earlier and had undergone total cystectomy. Therefore, a comprehensive immunohistochemical examination was necessary to confirm the diagnosis.

The immunohistochemical markers helped differentiate between various conditions, including poorly differentiated or undifferentiated gastric carcinoma, gastric neuroendocrine tumor, epithelioid gastrointestinal stromal tumor, leiomyosarcoma, paraganglioma, and urothelial carcinoma. The tumor cells showed positive staining for CKAE1/AE3, CK8/18, CK7, 34βE12, and GATA-3, while other antibodies were negative, excluding gastrointestinal stromal tumor, leiomyosarcoma, and paraganglioma. However, distinguishing between primary gastric adenocarcinoma and urothelial carcinoma is challenging. It has been noted that antibodies such as GATA3, CK7, 34βE12, and p63 are serving as potential markers for urothelial carcinoma, but these markers are not entirely specific and often require a combination to reach a definitive diagnosis (19). Both urothelial carcinoma and gastric adenocarcinoma can be positive for CK7, while urothelial carcinoma is typically positive for p63. In the present case, the absence of p63 made it challenging to distinguish primary gastric adenocarcinoma from metastatic bladder carcinoma. A careful review of the hematoxylin–eosin stained slices and immunohistochemical profile of the primary bladder tumor revealed that the tumor cells of stomach exhibited similar morphology with the bladder cancer. Additionally, the bladder cancer was also positive for GATA3, 34βE12, and CK7 while negative for p63. GATA3 and 34βE12 are relatively specific immunohistochemical markers for urothelial carcinoma, which played a crucial role in differentiation (20–22). In addition, no signs of atrophic gastritis, gastric intestinal metaplasia, or high-grade dysplasia were found in the gastric mucosa surrounding the cancer, which were often associated with primary early gastric cancer. Based on this comprehensive evaluation, the diagnosis of gastric oligometastatic carcinoma originating from invasive high-grade urothelial carcinoma of the bladder was confirmed. Regrettably, due to the high cost, NGS wasn’t performed on the bladder and gastric tumor. As an identical genetic profile may greatly increased the likelihood the gastric lesion is in fact a met of the bladder cancer.

In this case, a poorly differentiated or undifferentiated carcinoma infiltration reached 800 μm into the submucosa was found. If it had been primary gastric cancer, tumor differentiation degree and the infiltration depth would have exceeded the indications for ESD, necessitating radical gastrectomy. Because for poorly differentiated or undifferentiated cancers, the long diameter needs to be not exceeding 2 cm and the depth should not exceed the mucosal layer can ESD excision be performed. In the case of differentiated gastric cancer, when the invasion depth is less than 500 μm and the maximum long diameter does not exceed 3 cm, endoscopic submucosal dissection (ESD) resection can be considered (23). However, a comprehensive evaluation of the medical history and pathological features led to a diagnosis of oligometastatic bladder urothelial carcinoma metastasizing to the stomach rather than primary gastric cancer. Therefore, this patient does not need a radical operation on the stomach. The lesion was successfully removed by ESD with negative margins, sparing the patient from radical gastrectomy. The patient subsequently received four cycles of gemcitabine chemotherapy and has remained recurrence-free for 41 months. Traditionally systemic chemotherapy is the primary treatment for metastatic bladder cancer (5, 24). Nonetheless, studies by Jung et al. (25) suggest that in patients without absolute contraindications for surgery, gastric metastatic carcinoma may benefit from surgical intervention. The concept of oligometastatic disease, defined by single-organ involvement, ≤3 metastatic sites, metastatic lesions ≤5 cm in diameter, and the absence of liver metastases, has been increasingly applied across various malignancies. Several studies indicate that surgical resection of metastatic lesions in bladder cancer patients with oligometastatic disease can significantly improve survival outcomes. The overall 5-year survival rate for metastatic bladder cancer remains approximately 5% (26). In 1982, Cowles et al. first reported lung metastasectomy in six patients with metastatic bladder cancer, with four surviving beyond five years. Two subsequent studies reported 5-year survival rates of 33 and 28% for metastatic bladder cancer patients undergoing metastasectomy (27, 28). Therefore, patients with gastric oligometastatic lesions may benefit from local resection, though further research and data accumulation are still warranted in this area.

In summary, oligometastatic bladder urothelial carcinoma metastasizing to the stomach is extremely rare and prone to misdiagnosis. If the gastric tumor is misdiagnosed as a primary cancer, the error could have a detrimental impact on the patient’s quality of life. For a 70-year-old patient, undergoing a total bladder resection followed by radical gastric surgery poses a substantial challenge to the patient’s physical condition. Fortunately by integrating the patient’s medical history, the microscopic morphology and immunohistochemical results of both gastric tumors and bladder tumors, an accurate diagnosis was achieved, allowing for precise treatment with ESD surgery and avoiding the potential gastric functional impairment associated with radical surgery for a misdiagnosed primary gastric cancer. This case offers valuable insights into the pathological diagnosis and treatment decision-making for bladder cancer metastasizing to the stomach.

## Data Availability

The raw data supporting the conclusions of this article will be made available by the authors, without undue reservation.
